# The effect of standard-dose wintertime vitamin D supplementation on influenza infection in immunized nursing home elderly residents

**DOI:** 10.3325/cmj.2021.62.495

**Published:** 2021-10

**Authors:** Ana Godan Hauptman, Amarela Lukić-Grlić, Jasmina Vraneš, Milan Milošević, Alenka Gagro

**Affiliations:** 1 Division for Metabolic Diseases, Department of Internal Medicine, University Hospital Center Zagreb, Zagreb, Croatia; 2 Department of Clinical Microbiology, Children's Hospital Zagreb, Zagreb, Croatia; 3 University of Zagreb School of Medicine, Zagreb, Croatia; 4 Microbiology Service, Teaching Institute of Public Health “Dr. Andrija Štampar,” Zagreb, Croatia; 5 Department of Environmental Health, Occupational and Sports Medicine, Andrija Štampar School of Public Health, University of Zagreb, School of Medicine, Zagreb, Croatia; 6 Depatment of Pediatrics, Children’s Hospital Zagreb, Zagreb, Croatia; 7 Faculty of Medicine, Josip Juraj Strossmayer University, Osijek, Croatia

## Abstract

**Aim:**

To investigate whether three-month oral vitamin D supplementation (800 IU in drops) reduces the risk of influenza infection in elderly nursing home residents vaccinated against influenza.

**Methods:**

This cross-sectional observational study enrolled 97 participants (73.2% women) who received one dose of seasonal trivalent 2016-2017 influenza vaccine. The patients were randomized into an experimental group, which received vitamin D supplementation for three months starting on the day of vaccination, and a control group, which did not receive vitamin D supplementation. The primary outcome was the number of influenza infections laboratory-confirmed using a rapid point-of-care test based on nasal swabs collected during vitamin D supplementation. The secondary outcome was serum 25-hydroxyvitamin D level at the end of the study.

**Results:**

The mean age ±standard deviation was 78.5± 8.8 years. All participants had vitamin D deficiency at baseline. Twenty-three participants who developed signs of respiratory infections during the study were tested for influenza virus. Although the number of influenza-positive participants was lower in the group receiving vitamin D supplementation as compared with the control group (5 vs 12), this difference was not significant. Vitamin D supplementation failed to increase 25(OH)D levels after three months of supplementation.

**Conclusion:**

Elderly nursing home residents in Zagreb County have a significant vitamin D deficiency. The recommended national supplementation of 800 IU daily failed to lead to vitamin D sufficiency and did not reduce the risk of influenza infection among the vaccinated elderly.

Vitamin D is a fat-soluble vitamin with hormonal qualities, playing a well-documented role in bone health and homeostasis of calcium and phosphate. In addition, vitamin D deficiency is associated with various chronic aging-related noncommunicable diseases in the elderly, such as dementia, cardiovascular morbidity, diabetes, cancer, autoimmune diseases, and increased mortality (1,2). Recently, vitamin D deficiency has been linked to the susceptibility to and course of coronavirus disease 2019 (3).

Humans derive the most of vitamin D from endogenous synthesis in the skin under the influence of ultraviolet radiation, whereas less than 10% is derived from food. Regardless of its source, vitamin D is biologically active only after hydroxylation in the liver and kidney. Total vitamin D supply is assessed by the serum concentration of 25-hydroxyvitamin D (25(OH)D), with a half-life in plasma of about two weeks (4). A concentration below 75 nmol/L is considered vitamin D deficiency (5). The risk factors for vitamin D deficiency are older age, male sex, living at a high altitude, darker skin, lack of sun exposure, diet, and lack of regular vitamin D supplementation (6). Since 25(OH)D concentration depends primarily on the synthesis in the skin, in countries such as Croatia, this concentration is expected to be lowest during winter, which was confirmed in postmenopausal women (7).

In developed countries, the elderly are defined as people aged ≥65 years (8). This population is at risk of vitamin D deficiency due to a reduced capacity of vitamin D formation in the skin after sun exposure, reduced 25(OH)D hydroxylation in the kidney, and a decreased number of vitamin D receptors (9). Nursing home residents and frail persons are at a particularly high risk of vitamin D3 deficiency (10,11). Many countries, including Croatia, have recognized this problem and most of them recommend taking 700-800 IU of vitamin D per day in order to improve bone health (12-14).

People of all ages are susceptible to influenza infection, but complications such as secondary infections, exacerbations of chronic comorbid conditions, and death are more prevalent in the frail elderly, particularly nursing home residents (15). The 2010 Cochrane review on influenza vaccination in the elderly by Jefferson et al confirmed vaccine safety, but failed to demonstrate its effectiveness and challenged the ongoing efforts to vaccinate the elderly (16). However, a more recent meta-analysis of the same clinical studies showed that influenza vaccine reduced the risk of infection, as well as of influenza-related disease and mortality in the elderly (17). Although the impact of influenza vaccine was evaluated with different outcome measures, certain outcomes, such as laboratory-confirmed influenza, are considered more specific in comparison with seroconversion to the circulating virus strain (18).

The immunogenicity of influenza vaccine in the elderly is considerably lower (30-40%) than in their younger counterparts (70-90%) (19). Nursing home residents are especially vulnerable given their immunosenescence and multimorbidity (20). A chronic antigenic stimulus important for immunosenescence is cytomegalovirus (CMV) infection, which usually leads to asymptomatic infection in healthy people. Namely, the majority of the elderly have antibodies against this virus, reflecting latent infection. Latent CMV infection is proposed to further enhance aging-induced changes, primarily of T-cell immunity, which increases the risk of respiratory viral infections and decreases the responsiveness to influenza vaccination in the elderly (21).

Based on *in vitro* data on modulatory actions of 25(OH)D on immune cells and several epidemiologic studies, an adequate 25(OH)D level could protect against influenza infections (22). Other micronutrients, such as zinc, selenium, and vitamin C have also recently been shown to protect against acute respiratory infections (23). However, a recent systematic review showed no significant impact of vitamin D deficiency on immunogenic response to influenza vaccination, although possible strain-specific differences were identified (24). Data on the effect of vitamin D supplementation on the incidence of influenza infection in the influenza-vaccinated elderly are lacking. Therefore, the aim of this study was to investigate whether three-month oral vitamin D supplementation (800 IU in drops) reduces the risk of influenza infection in elderly nursing home influenza-vaccinated residents.

## Patients and methods

This prospective randomized study enrolled 116 Croatian individuals living in two nursing homes in Zagreb County. The study was conducted from October 2016 until August 2017. The ethical approved was obtained from the Ethics Committees of the nursing homes and the Ethics Committee of the University of Zagreb, School of Medicine (380-59-10106-20-111/170). Participants or their legal guardians signed the informed consent for study participation. The inclusion criteria were age ≥65 years, minimum length of stay in the nursing home of 12 months before enrolment, and frailty assessed by the Clinical Frailty Scale. This scale evaluates specific domains including comorbidity, function, and cognition to generate a frailty score ranging from 1 (very fit) to 9 (terminally ill), where a person with a score ≥5 is considered frail (25). The exclusion criteria were terminal illness, refusing participation, and malignant disease. Among 116 nursing home residents, 97 met the inclusion criteria. In addition to demographic data such as age and sex, the data on the following parameters were collected: comorbidities (chronic obstructive pulmonary disease [COPD], coronary disease, heart failure, diabetes mellitus, stroke, dementia), using ≥5 drugs, and body mass index (BMI) calculated as weight/height^2^ (kg/m^2^) and classified as underweight (BMI <18.5), normal weight (BMI 18.5-24.99), overweight (BMI 25-29.99), and obesity (BMI ≥30).

The participants included in the study were vaccinated against influenza with the seasonal trivalent vaccine containing A/California/7/2009 (H1N1) pdm09, A/Hong Kong/4801/2014(H3N2), and B/Brisbane/60/2008 viruses. Following vaccination, they were randomized into two groups using a publicly available program (Urbaniak GC, Plous S. (2013). Research Randomizer, version 4.0; retrieved on June 22, 2013, from http://www.randomizer.org/). The experimental group started vitamin D supplementation (800 IU in drops) in the morning on the day of immunization under supervision of a nurse and continued it for three months. The control group did not receive vitamin D supplementation. Vitamin D supplementation was administered according to the respective national recommendations (14).

Throughout the three winter months, the participants were monitored by nursing home employees (certified nurses and once a day by a physician) for the signs of febrile acute respiratory viral illness. In participants presenting with febrile respiratory illness, samples of the posterior nasopharynx were obtained via nylon flocked swab in addition to blood samples for whole blood analysis, C-reactive protein, serum immunoglobulin (IgM, IgG, and IgA) levels, CMV-specific IgM and IgG levels (ETI-Cytok M/ETI-Cytok G; DiaSorin, Saluggia, Italy), and biochemical urine analysis. Based on these findings, along with clinical examination, breathing frequency, and oxygen saturation (determined by percutaneous transcutaneous oximetry) findings, a diagnosis of upper or lower respiratory infection was established by an attending physician. Nasopharyngeal swabs were obtained in symptomatic participants within the first five days of acute respiratory illness, placed in transport media, and stored in a refrigerator until transfer to the microbiology laboratory within 24 hours.

The primary outcome was the number of laboratory-confirmed influenza infections. This number was ascertained by detecting the presence of respiratory viruses in nasopharyngeal swabs using the mariPOC® respi test for influenza-like illnesses (ArcDia International Qy Ltd., Turku, Finland), which detects eight respiratory viruses (influenza A virus, influenza B virus, respiratory syncytial virus, human coronavirus OC43, human metapneumovirus, human bocavirus, parainfluenza virus type 1-3, and adenovirus) according to the manufacturer's instructions. The performance of mariPOC® assay in comparison with polymerase chain reaction declared by the manufacturer is as follows: sensitivity 92.3% for influenza A and 88% for influenza B; and specificity 99.8% for influenza A and 100% for influenza B.

The secondary outcome was the determination of 25(OH)D level at baseline and after three months. Fasting blood samples were drawn in the morning between 8.00 and 10.00 a.m. and kept at -20°C for a maximum of six months prior to analysis. Serum 25(OH)D was determined using the LIAISON® 25 OH Vitamin D TOTAL Assay (DiaSorin Inc., Stillwater, MN, USA) at baseline and after three months. The measuring range of this assay is 4.0-150 ng/mL, with an intra-assay precision of 7%. 25(OH)D levels were categorized as follows: very severe deficiency <12.5 nmol/L; severe deficiency 12.5-24 nmol/L; moderate deficiency 25-49 nmol/L; minor deficiency 50-74 nmol/L; and normal level 75-175 nmol/L.

### Statistical analysis

The first aim was to determine the number of participants and their age, sex, body mass index, Clinical Frailty Scale score, comorbidities, concomitant medications, and 25(OH)D concentrations. The second aim was to calculate the prevalence and severity of vitamin D deficiency. The third aim was to identify the factors contributing to 25(OH)D increase after three-month supplementation in the analysis adjusted for age, sex, medications used, and Clinical Frailty Scale score. The difference in 25(OH)D levels between the baseline and after three months was assessed by the Wilcoxon test. The Fisher exact test was used to assess the difference in clinical and socio-demographic variables in participants with confirmed influenza respiratory viral disease. Ordinary least square regression adjusted for age, sex, medications used, and Clinical Frailty Scale score was used to assess the effect of vitamin D supplementation on vitamin D concentration increase after three months. The level of significance was set at p<0.05. The statistical analysis was performed with SPSS, version 25.0 (IBM Corp., Armonk, NY, USA).

## Results

Out of 97 participants enrolled, 91 completed the study. Four individuals did not undergo the second blood sampling since they died from their chronic illness or influenza infection complications. Two participants left the nursing home to live with their families (Table 1).

**Table 1:** Sociodemographic and clinical characteristics of study participants (N=97)

Characteristic	
Female sex; n (%)	71 (73.2)
Age (years); mean ± standard deviation (SD)	78.5±8.8
Body mass index (BMI, weight/height^2^ in kg/m^2^); mean ± SD	22.4±2.8
underweight (<18.5); n (%)	2 (2.1)
normal weight (18.5-24.99); n (%)	73 (75.2)
overweight (25-29.99); n (%)	22 (22.7)
obesity (≥30); n (%)	0 (0)
Clinical Frailty Scale score; median (interquartile range [IQR])	6.0 (5.0-6.0)
Cardiovascular comorbidity; n (%)	87 (89.7)
Respiratory comorbidity (chronic obstructive pulmonary disease); n (%)	38 (39.2)
Diabetes mellitus; n (%)	20 (20.6)
Heart failure; n (%)	10 (10.3)
Cerebrovascular disease; n (%)	38 (39.2)
Dementia; n (%)	43 (44.3)
Autoimmune diseases; n (%)	22 (22.7)
The number of concomitant medications; median (IQR)	5.0 (4.0-7.0)
Vitamin D supplementation; n (%)	41 (42.3)
Death outcome; n (%)	4 (4.1)

**Figure 1:**
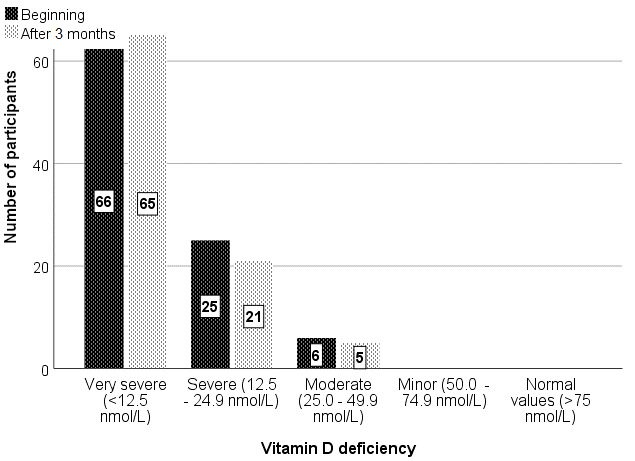
The number of participants with vitamin D deficiency at baseline and after three months of vitamin D supplementation.

At study entry, all participants had lower 25(OH)D levels than those currently recommended for optimal health. In addition, the prevalence of very severe and severe vitamin D deficiency was very high (94%) (Figure 1).

**Figure 2:**
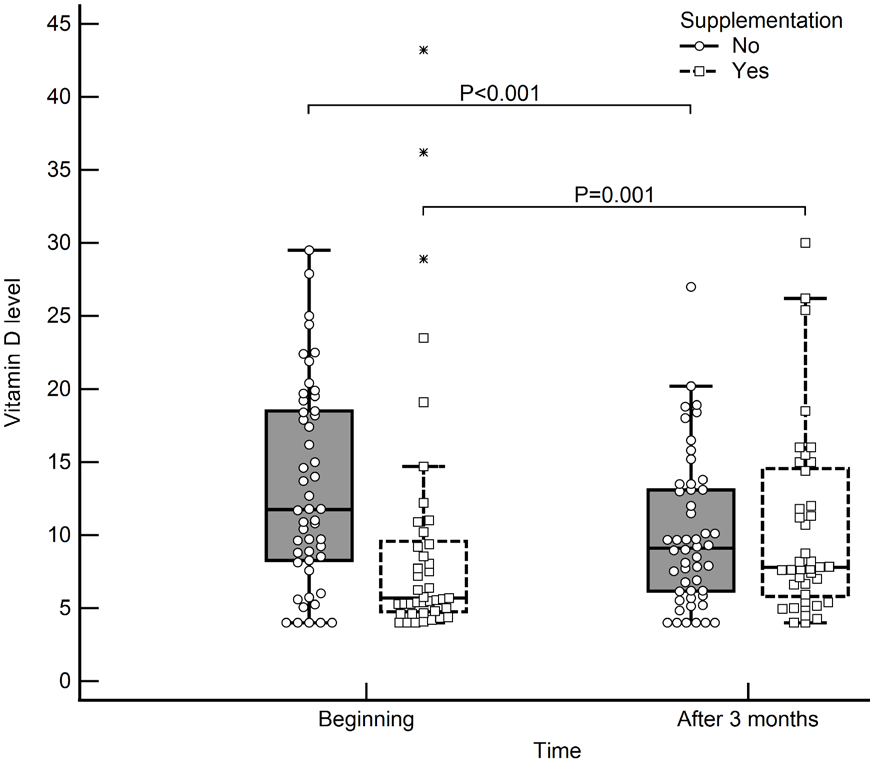
Serum 25(OH)D levels in elderly nursing home residents who received D supplementation for three months and in controls.

Following randomization, the experimental group (n=41) received daily vitamin D supplementation (800 IU in drops *per os*) in the morning for three months. The control group (n=57) received no vitamin D supplementation. Vitamin D supplementation increased the 25(OH)D levels in 26 (63.41%) experimental group participants (Figure 2). Vitamin D concentration significantly decreased in the group without vitamin D supplementation (from 11.4 [7.7-18.3] to 9.1 [6.1-13.2] ng/mL, p<0.001), whereas it increased from 5.7 (4.7-9.8) to 7.8 (5.7-14.7) ng/mL (p=0.001) in the group receiving vitamin D supplementation. However, after three months none of the participants having received vitamin D supplementation reached the recommended 25(OH)D levels.

We also assessed the impact of age, sex, number of medications, and Clinical Frailty Scale score on the effect of vitamin D supplementation on serum 25(OH)D level increase. Supplementation was positively associated with serum 25(OH)D increase even after adjustment for age, sex, number of medications, and Clinical Frailty Scale score (β=0.50, 95% CI 0.28-0.56, p<0.001) (Table 2).

**Table 2:** The effect of vitamin D supplementation on serum vitamin D increase after three months of supplementation (adjusted for age, sex, medications used and Clinical Frailty Scale score); ordinary least square regression

	Standardized coefficients	95% confidence interval		P
	beta	lower	upper	
Supplementation	0.50	0.28	0.56	<0.001
Age (years)	0.15	0.00	0.02	0.100
Female sex	0.01	-0.14	0.17	0.879
Number of concomitant medications	0.14	-0.01	0.07	0.130
Clinical Frailty Scale score	0.17	-0.01	0.23	0.065

Twenty-three participants who developed signs of respiratory infections during the study were tested for influenza virus using a point-of-care test for respiratory viral infections. Seventeen participants tested positive for influenza A virus. Participants with influenza infection had a higher incidence of death (p<0.001) and heart failure (p=0.014). Although the number of influenza-positive participants was lower in the group receiving vitamin D supplementation than in the control group (5 vs 12), the difference was not significant (Table 3).

**Table 3:** Comparison of socio-demographic and clinical variables between influenza-positive and influenza-negative participants

		Influenza-positive				*P* (Fisher exact test)
		no		yes		
		n	%	n	%	
Sex	male	21	26.3	5	29.4	0.770
	female	59	73.8	12	70.6	
Vitamin D supplementation	no	44	55.0	12	70.6	0.288
	yes	36	45.0	5	29.4	
Death outcome	no	80	100.0	13	76.5	<0.001
	yes	0	0.0	4	23.5	
Chronic obstructive pulmonary disease	no	52	65.0	7	41.2	0.100
	yes	28	35.0	10	58.8	
Cardiovascular comorbidity	no	8	10.0	2	11.8	1.000
	yes	72	90.0	15	88.2	
Diabetes mellitus	no	66	82.5	11	64.7	0.110
	yes	14	17.5	6	35.3	
Heart failure	no	75	93.8	12	70.6	0.014
	yes	5	6.3	5	29.4	
Cerebrovascular disease	no	48	60.0	11	64.7	0.790
	yes	32	40.0	6	35.3	
Dementia	no	44	55.0	10	58.8	1.000
	yes	36	45.0	7	41.2	
Autoimmune disease	no	61	76.3	14	82.4	0.755
	yes	19	23.8	3	17.6	

A total of 79 (81.4%) participants tested positive for IgG against CMV at baseline, while none had CMV-specific IgM antibodies. Furthermore, none had a decrease in major serum immunoglobulins (IgM, IgG, and IgA) at the time of randomization. Sociodemographic variables, changes in vitamin D concentrations, and clinical evidence for respiratory infection did not significantly correlate with the degree of immunosenescence as assessed by serum IgG against CMV (results not shown).

## Discussion

Our study showed that residents of two nursing homes had a very high prevalence (94%) of very severe and severe forms of vitamin D deficiency irrespective of age, sex, number of medications, and Clinical Frailty Scale score. This prevalence rate significantly exceeded the prevalence of vitamin D deficiency (<30 nmol/L) observed in postmenopausal women (14.2%) reported in the only previously published study on vitamin D status in our country (7). This discrepancy was expected as our cohort included older participants characterized by significant frailty, reduced mobility, and consequential reduction of sun exposure, as a well-documented risk for vitamin D deficiency. However, the rate of severe deficiency in our study was similar to those reported in some other European countries (26,27).

Although we found an increased vitamin D concentration among the participants having received the recommended vitamin D supplementation, these values were critically below the recommended values in our country, thus necessitating a different supplementation approach in this vulnerable population. More recent Croatian recommendations, published after this study had been approved by the Ethics Committee, recommend higher doses of vitamin D (1500-2000 IU/day) for patients at risk of vitamin D deficiency and therefore supporting the Endocrine Practice Guidelines Committee guidelines instead of those issued by the American Institute of Medicine (800 IU/day) (28). Indeed, 2000 IU/daily of vitamin D achieved 25(OH)D3 values of >80 nmol/L in most nursing home residents (29). However, these authors did not determine the baseline level of 25(OH)D, so it is not clear if this approach could be used in all nursing home residents regardless of the baseline 25(OH)D levels.

An alternative approach using the loading dose of 200,000 IU and maintenance dose (100,000 IU) administered every 13 weeks also failed to reach and maintain the vitamin D concentration of 75-220 nmol/L in nursing home residents (30). Another study suggested that an individualized loading dose of vitamin D be given to those with severe 25(OH)D deficiency (31). At the time of the study, only one vitamin D preparation exclusively in the form of drops available for oral treatment was covered in total by the national health insurance, so we were not able to prescribe alternative forms of vitamin D. It should also be stressed that no consensus has been reached on the optimal 25(OH)D level and vitamin D deficiency. Whatever the threshold selected, there is a consensus on vitamin D deficiency being associated with poor health in the elderly, particularly in nursing home residents (32).

A recent systematic review of 26 studies showed a highly variable incidence (from 1.21% to 85.2%) of acute respiratory infections among older adults in nursing homes. Infections caused by influenza and respiratory syncytial viruses are most common, but other viruses (eg, parainfluenza, human metapneumovirus, and rhinoviruses) are also associated with infection-dependent morbidity and mortality (20). Many vulnerable people with underlying chronic diseases in nursing homes share common spaces for daily activities, and guidelines for prevention of respiratory infections are difficult to follow, which may lead to spread of infections. Furthermore, daily visitors and the nursing home staff can introduce viral infections from the community (33). Given the limited treatment options and consequences of the most common viral infections in the elderly, early detection of infections and vaccination before the influenza season, which in Croatia is free for the elderly, are key measures to prevent morbidity and mortality, reduce the use of unnecessary antibiotics, and rationally implement isolation measures (34,35).

During our study, 23 (25%) of 91 participants having completed the study had a lower respiratory tract viral infection during winter months, as revealed by physician’s clinical examination, breathing frequency, oxygen saturation determined by percutaneous transcutaneous oximetry, and laboratory testing. Of eight respiratory viruses tested in these 23 participants by a point-of-care test, we detected influenza A virus in 17 participants. Given the lower sensitivity of viral-antigen based point-of-care testing in comparison with molecular methods (36), it cannot be ruled out that other participants with respiratory infection had either influenza or some other respiratory viral infection.

The number of influenza-positive participants was lower in the group receiving vitamin D supplementation than in the control group; however, this difference was not significant. Furthermore, two participants who had influenza and died due to complications had received vitamin D supplementation. These results indicate that in our study vitamin D supplementation demonstrated no anti-infective or anti-inflammatory effect, as recently discussed (37). More recent national recommendations for vitamin D supplementation in the groups at risk, which include the frail elderly nursing home residents (28), as well as the findings of a recent meta-analysis, indicate that individuals with profound vitamin D deficiency and those not receiving bolus doses of vitamin D benefit most from vitamin D supplementation (38). Therefore, it is tempting to speculate that this approach would be more effective in preventing influenza infection in vaccinated elderly nursing home residents.

The physiological processes of immunosenescence, as well as comorbidities, contribute to the fragility of the elderly and to the increased risk and adverse outcome of viral respiratory infections (20). Humoral immunity, which plays an important role in preventing influenza virus transmission and infection, declines with age (39). In our study sample, none of the participants had decreased levels of serum immunoglobulins at randomization, so we excluded the possible contribution of an age-associated deficit of humoral immunity as a risk factor for respiratory viral infection (data not shown). However, a recent study demonstrated that vitamin D supplementation induced a higher plasma level of transforming growth factor beta as an indicator of tolerogenic immune response following influenza vaccination without improving antibody production and thereby influenced predominantly cell-mediated immunity (40).

Cytomegalovirus is considered as an important contributor to chronic antigenic stimulation important for immunosenescence (41). Influenza vaccine inefficiency also has been linked to the reactivation of CMV infection in the elderly (42). We observed no link between CMV seropositivity and the lack of influenza-vaccine protection either in vitamin D supplemented participants or in the control group.

We showed for the first time that the great majority of elderly nursing home residents in Zagreb County had a severe vitamin D deficiency and that a three-month supplementation with 800 IU of cholecalciferol daily according to the current national guidelines failed to increase 25(OH)D levels to the recommended range. Our study demonstrated that 800 IU/day of oral vitamin D supplementation during winter did not prevent influenza infections in the vaccinated elderly.
